# Somatosensory innervation of the oral mucosa of adult and aging mice

**DOI:** 10.1038/s41598-018-28195-2

**Published:** 2018-07-02

**Authors:** Yalda Moayedi, Lucia F. Duenas-Bianchi, Ellen A. Lumpkin

**Affiliations:** 1 Department of Physiology and Cellular Biophysics, Columbia University, New York, NY 10032 USA; 2 SPURS Biomedical Research Program, Department of Physiology and Cellular Biophysics, Columbia University Medical Center, New York, NY 10032 USA; 3 Department of Dermatology, Columbia University, New York, NY 10032 USA; 4 Program in Neurobiology and Behavior, Columbia University, New York, NY 10032 USA

## Abstract

Oral mechanoreception is implicated in fundamental functions including speech, food intake and swallowing; yet, the neuroanatomical substrates that encode mechanical stimuli are not well understood. Tactile perception is initiated by intricate mechanosensitive machinery involving dedicated cells and neurons. This signal transduction setup is coupled with the topology and mechanical properties of surrounding epithelium, thereby providing a sensitive and accurate system to detect stress fluctuations from the external environment. We mapped the distribution of anatomically distinct neuronal endings in mouse oral cavity using transgenic reporters, molecular markers and quantitative histomorphometry. We found that the tongue is equipped with an array of putative mechanoreceptors that express the principal mechanosensory channel Piezo2, including end bulbs of Krause innervating individual filiform papillae and a novel class of neuronal fibers innervating the epithelium surrounding taste buds. The hard palate and gums are densely populated with three classes of sensory afferents organized in discrete patterns including Merkel cell-neurite complexes, Meissner’s corpuscles and glomerular corpuscles. In aged mice, we find that palatal Merkel cells reduce in number at key time-points that correlate with impaired oral abilities, such as swallowing and mastication. Collectively, this work identifies the mechanosensory architecture of oral tissues involved in feeding.

## Introduction

The oral cavity is responsible for a variety of complex behaviors, including feeding and speech. These behaviors are governed by chemosensory and somatosensory neurons, which converge in the mouth to transduce chemical and physical stimuli. For example, thermal and mechanical qualities of food are important components of flavor determination during food choice^1,2^. Mechanical qualities such as grittiness, stickiness, viscosity, hardness and greasiness are used as proxies to determine freshness and nutritional content of foods^3^. Food structure, and thus, texture perception also impacts caloric intake^2,4,5^. As food is chewed, the texture of the bolus determined through a psychophysical interpretation of its structural and mechanical properties^6,7^ modulates aspects of bite force^8,9^, tongue position, and determination of appropriate swallow time^7^. Mechanical inputs on the back of the throat then trigger and perpetuate the swallow reflex^10^. Despite their importance in feeding, little is known about somatosensory neurons in the oral cavity that encode physical qualities, such as texture. The environment of the oral cavity poses a particularly complex location for the study of texture perception, as it is composed of multiple epithelial surfaces (e.g. tongue, hard palate, gingiva) with differences in tissue compliance and neuronal structures. In order to understand the physiological basis for texture in feeding, it is necessary to analyze the somatosensory substrates in these tissues.

During aging, the ability to detect the mechanical properties of foodstuffs declines. Reduced mechanosensitivity can have profound implications for quality of life that recapitulate elderly oral pathologies; in fact, damage to the lingual branch of the trigeminal nerve results in tongue biting, inability to position foods in the mouth, burning mouth syndrome, speech alterations and perceptual changes in food quality^11^. Amongst the elderly, age-related reductions have been reported in two-point discrimination abilities in tongue, cheeks and lips^12^; touch threshold detection in the cheeks, tongue and anterior palate^13^; and tongue vibrotactile sensitivity, a correlate of speech production abilities^14^. In addition to these, the ability to discriminate shapes, a correlate of masticatory abilities termed stereognosis, declines with age^15–18^. Few studies have addressed the neural correlates of sensory decline in the oral cavity. In histology, decreases in overall innervation density to human gingiva have been identified with aging^19^. Similarly, in the aging murine hard palate, reductions in complexity and size of Meissner’s corpuscles have been reported^20^. However, alterations in other mechanosensory structures have not been investigated. The alterations in neuronal architecture that underlie particular aspects of age-related tactile decline are an open research topic.

Conserved features of cutaneous somatosensation can guide the study of oral tissues. In the skin, mechanosensory cells detect an array of physical stimuli including pressure and vibrations. Somatosensory neurons that encode tactile stimuli have anatomically specialized peripheral terminals, termed end organs, that shape the neuron’s response properties to sensory stimuli. Several distinct cutaneous end organ subtypes discriminate unique aspects of touch. For example, encapsulated corpuscles, such as Meissner’s and Pacinian corpuscles, detect texture and vibrations of different frequencies^21^. Merkel cell-neurite complexes are required for shape discrimination and reporting sustained pressure^21^. Cutaneous mechanosensory endings can be localized in tissues based on anatomical structure combined with molecular properties such as expression of the myelinated neuron marker Neurofilament-Heavy (NFH), expression of the principal mechanosensory protein Piezo2^22^, and uptake of the styryl dye FM1-43^23^. On the other hand, unmyelinated free nerve endings sense nociceptive, thermal, and chemical stimuli and can be visualized by expression of peripherin^24^. In the oral cavity, multiple classes of mechanosensitive cells and neurons have been reported including: Merkel cell-neurite complexes, encapsulated corpuscles and free nerve endings^25–31^. These reports have relied primarily on ultrastructural electron microscopy and have resulted in conflicting results on the distribution and densities of presumptive mechanoreceptors in the oral cavity. A comprehensive analysis of mechanoreceptors in the murine oral cavity using modern histological methods is thus lacking. These studies are essential for understanding how somatosensory alterations affect feeding pathologies.

Here we present a systematic histological analysis using modern molecular biology tools to analyze the anatomical and molecular diversity of somatosensory receptors in the oral cavity in young adult and aged mice.

## Results

### Sensory innervation of the lingual mucosa

**Figure 1:**
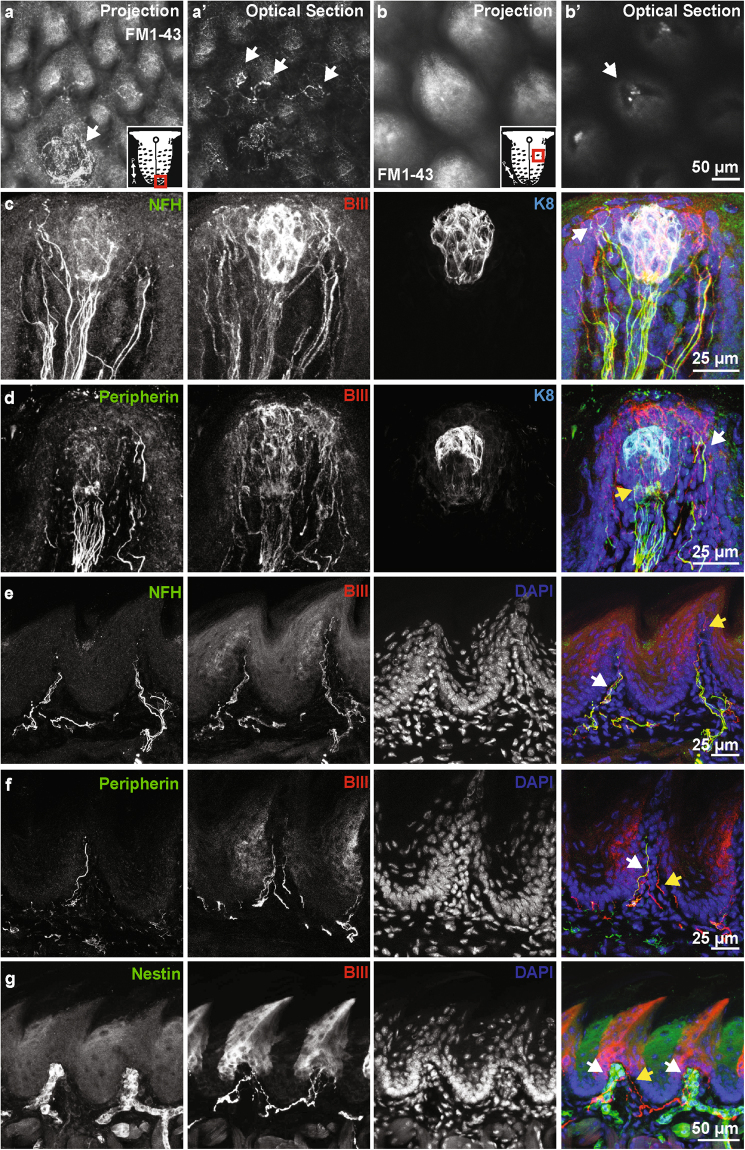
The lingual mucosa is innervated by multiple neuronal classes. (**a**) Whole mount imaging of FM1-43 labeling of neuronal afferents in the tongue. A projection shows neuronal endings surrounding a fungiform papillae in the anterior tongue. An optical section through this image reveals neuronal endings innervating individual filiform papillae (**a**’, arrows). Schematic shows tongue region represented. (**b**) Whole mount imaging of filiform papillae in posterior tongue. An optical section through the base of the papillae (**b’**) reveals neuronal afferents innervating the core of individual papillae (arrow). Schematic shows tongue region represented. (**c**) Neurofilament heavy (NFH) positive and negative fibers innervate fungiform papillae. NFH fibers extend into the epidermis overlying the taste cells (arrow). (**d**) Peripherin+ fibers also enter the fungiform papillae, these primarily associate with taste cells (yellow arrow, K8), but also extend around to the overlying epithelium (white arrow). (**e**) Filiform papillae are innervated with NFH+ afferents (white arrow). Note other afferents extend into the apical regions of the papilla (yellow arrow). (**f**) Peripherin+ (white arrow) and Peripherin- (yellow arrow) afferents are also present in the filiform papilla, demonstrating that multiple neuronal subtypes innervate these non-taste papillae. (**g**) A subset of neuronal endings in the filiform papillae are surrounded by Nestin+ cells.

To identify sensory innervation in the tongue, we first analyzed uptake of FM1-43, a styryl dye that can enter sensory cells through non-selective cation channels often associated with mechanosensory afferents^23^. Innervation was analyzed in two classes of lingual papillae, the fungiform taste papillae which are speckled throughout the anterior two-thirds of the tongue, and the filiform non-taste papillae, which comprise the majority of the tongue surface. A collar of FM1-43+ neurons surrounded fungiform taste buds and FM1-43+ afferents were identified in tight association with filiform papillae (Fig. 1a,b and Supplemental Movie 1). In optical sections, we found that neuronal endings are associated with individual filiform papillae (Fig. 1a’,b’). Collectively, these data suggest that both fungiform and filiform papillae are equipped with neuronal endings innervating non-taste structures.

To identify the molecular classification of neurons innervating lingual papillae, we employed section immunohistochemistry with neuronal markers associated primarily with small-diameter, unmyelinated neurons (peripherin) and medium- and large-diameter, myelinated neurons (NFH). Antibodies against keratin 8 (K8) and β-III tubulin (βIII) were used to identify taste cells and all neurons, respectively. Fungiform papillae were surrounded by NFH+ afferents that extended into the surrounding epithelium adjacent to K8+ taste buds (Fig. 1c). Peripherin+ neuronal afferents also extended into fungiform papillae into the overlying epithelium and in association with taste cells as presumptive gustatory afferents (Fig. 1d). We next analyzed non taste-associated papillae in the tongue. Filiform papillae were innervated by both NFH+ and peripherin+ afferents (Fig. 1e,f). Interestingly, some neurons extended processes into the apical portions of filiform papillae, perhaps providing additional access to the external environment as thermal or chemical receptors (Fig. 1e). As previously described in cat tongue^29^, all filiform papillae were innervated with encapsulated end bulbs of Krause, visualized by Nestin+ Schwann cells surrounding afferents (Fig. 1g). Collectively, these data suggest that individual filiform and fungiform papillae are innervated by multiple neurons of different classes, likely transducing different sensory sub-modalities.

Merkel cells have been reported in the tongues of primates, birds, reptiles and amphibians^32–35^ but have not been identified in rodents. Thus, we attempted to localize Merkel cells within the murine lingual epithelium. To this end, we employed reporter mouse lines that express genetically encoded markers under the control of *Atoh*1, a basic helix-loop-helix transcription factor that is essential for Merkel-cell development and the earliest known selective marker for Merkel cells in skin^36,37^. The location and density of Merkel cells in the lingual mucosa was first mapped using *Atoh1*^*LacZ*/+^ mice^37^. In the *Atoh1*^*LacZ*/+^ targeted allele, the endogenous *Atoh1* coding region is replaced with *LacZ*; however, no haploinsufficiency phenotypes have been identified in *Atoh1*^*LacZ*/+^ mice^38^. Whole mount X-gal staining of *Atoh1*^*LacZ*/+^ tongues revealed diffuse staining in a pattern similar to taste bud locations (Supplemental Fig. 1a,b). X-gal staining was absent in wild-type (WT) littermates lacking the *LacZ* allele, indicating that the staining is specific for *Atoh1* locus expression. To identify cellular localization of *Atoh1*, alternating tissue sections were analyzed spanning entire tongues of *Atoh1*^*tm4*.*1Hzo*^ mice, which express an Atoh1-green fluorescent protein (GFP) fusion protein^39^. K8 labels both mature Merkel cells and taste cells, and therefore the use of transgenic mice is essential to identify bona fide Merkel cells in this tissue^40^. Only a single GFP^+^K8^+^ cell was identified in three tongues examined in this manner. This cell was located within a fungiform papilla adjacent to the taste bud and was of an atypical shape (Supplemental Fig. 1c). Interestingly, in these sections low and punctate expression of GFP staining in taste buds was identified, which likely correlates with X-gal staining observed in *Atoh1*^*LacZ*/+^ taste buds. These data indicate that Merkel cells are not a major sensory cell in the mouse tongue.

### Sensory innervation of the gingiva and palatal mucosa

**Figure 2:**
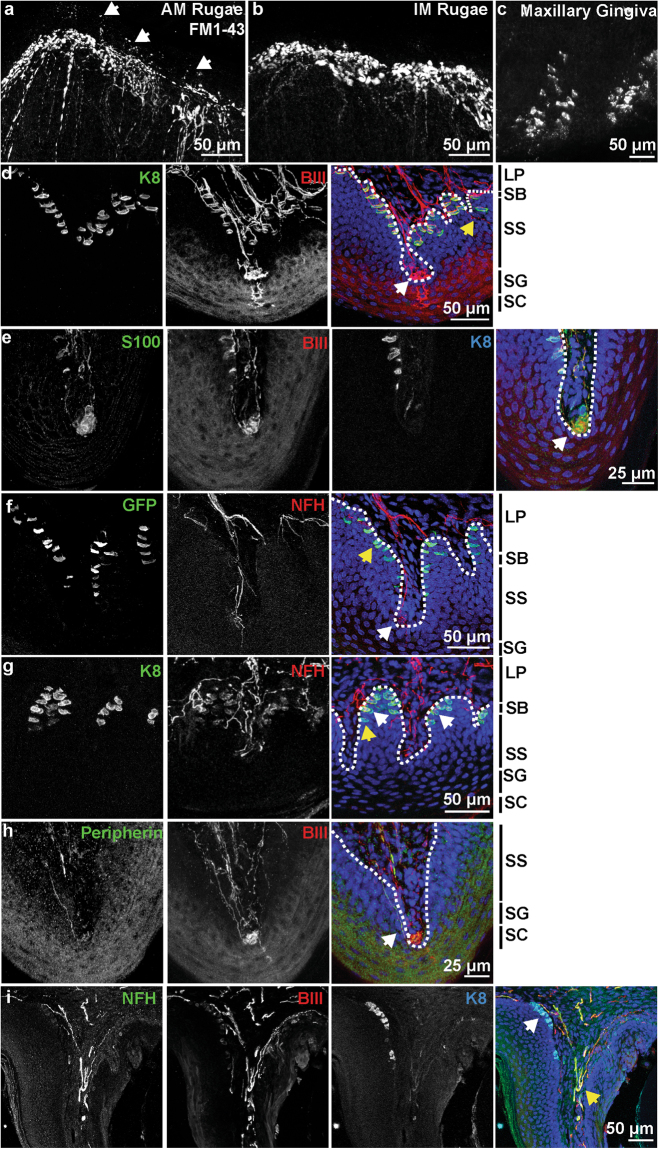
Maxillary epithelia are rich in neuronal innervation and Merkel cells. (**a**) Whole mount imaging of FM1-43 labeling in an antemolar ruga of the hard palate. Dense innervation lines the ridge of the antemolar ruga, with ultraterminals jutting into the epithelium of the ruga (arrows). (**b**) FM1-43 labels dense neuronal endings in the ridges of intermolar ruga. (**c**) FM1-43 labels Merkel cell clusters in maxillary gingiva. (**d**)Innervation of hard palate rugae was visulaized with βIII tubulin and K8. Glomerular-type endings were embedded in epithelial pegs (white arrow), often complemented with an ultra-terminal (asterisk). Free nerve fibers innervated the epithelium (yellow arrow). K8 staining showed Merkel cells densely packed along the epithelial-lamina propria junction. (**e**) Glomerular endings (arrow) associated with end-terminal Schwann cells, visualized by S100 staining. (**f**) NFH revealed neuronal endings with Meissner’s morphology (white arrow). This was also associated with an ultra-terminal (asterisk). Atoh1-GFP+ Merkel cells were found lining the epithelial ridges. Yellow arrow denotes a Merkel cell that is not innervated by a NFH+ afferent. (**g**) Coronal section of a hard palate ridge shows K8+ Merkel cells organized in epithelial pegs. Merkel cells innervated by NFH+ afferents (white arrow) as well as Merkel cells without NFH+ neuronal connections (yellow arrow) are denoted. (**h**) Peripherin+ endings were found innervating palatal ridges (arrow). These did not appear to be a large component of the glomerular-type endings. Merkel cells line exterior aspect of maxillary gingiva mucosa (white arrow), visualized by coronal sections and immunohistochemistry. NFH+ neurons innervate the gingiva as well (yellow arrow); however, these did not appear to form organized end organs. (LP: lamina propria, SB: stratum basale, SS: stratum spinosum, SG: stratum granulosum, SC: stratum corneum, Dotted line marks LP-SB border).

Somatosensory innervation of maxillary tissue was analyzed using FM1-43 uptake and whole mount imaging. As previously reported^27^, dense innervation in the palatine rugae was identified by FM1-43 (Fig. 2a,b). Innervation was present in the apical ridges of both antemolar and intermolar rugae. Frequent apical neuronal extensions, deemed ultra-terminals^41^, were found jutting above neuronal clusters (Fig. 2a, arrows). In the gingival mucosa, cells with the morphology of Merkel cells labelled with FM1-43, and FM1-43+ neuronal endings were sparse (Fig. 2c). Thus, the palatal mucosa is densely packed with FM1-43+ endings concentrated in rugal ridges while the gingiva is lined with Merkel cells.

We next performed multiplex immunohistochemistry on hard palate sections to classify neuronal afferents innervating rugae. Glomerular endings (Fig. 2d, white arrow) were found throughout the palatine mucosa and were classified by having dense bundles of neurons. As previously reported^42^, glomerular endings in the hard palate were surrounded by S100+ terminal Schwann cells (Fig. 2e). Meissner’s corpuscles, visualized by NFH^22^ (Fig. 2f, arrow), were found in the lamina propria and were distinguished from glomerular corpuscles based on their elegant neuronal endings comprised of several turns of one or more NFH+ fibers. Both glomerular endings and Meissner’s corpuscles were often associated with an ultra-terminal that projected to the superficial layers of the stratum spinosum and stratum granulosum (Fig. 2d,f, asterisk). Merkel cells in palatine rugae were heavily innervated by βIII+ neuronal afferents (K8, Fig. 2d), collectively forming Merkel cell-neurite complexes. Merkel cells were Atoh1+ (GFP, Fig. 2f), and were comprised of cells either contacted by NFH+ afferents (Fig. 2g, white arrow), or uncontacted (Fig. 2f,g, yellow arrow). Free nerve endings were located in the epithelium with terminations in the basal layers of the stratum spinosum (Fig. 2d, yellow arrow). Peripherin+, small-diameter afferents were found adjacent to glomerular endings (Fig. 2g). These endings were distinct from ultra-terminals in that they were not found in proximity to a corpuscular structure, terminated in basal layers of the epithelium, and tended to be on the lateral sides of rugae.

In the maxillary gingiva, K8+ Merkel cells were identified on the external aspects of the molars (Fig. 2i, white arrow). NFH+, βIII+ neuronal endings were found coursing through the lamina propria; however, no organized end-organ structures were apparent (Fig. 2i, yellow arrow). Thus, an array of putative somatosensory afferents innervates the murine hard palate while the maxillary gingiva is primarily innervated by Merkel cell-neurite complexes.

### Mechanosensory neurons of the oral epithelium

**Figure 3:**
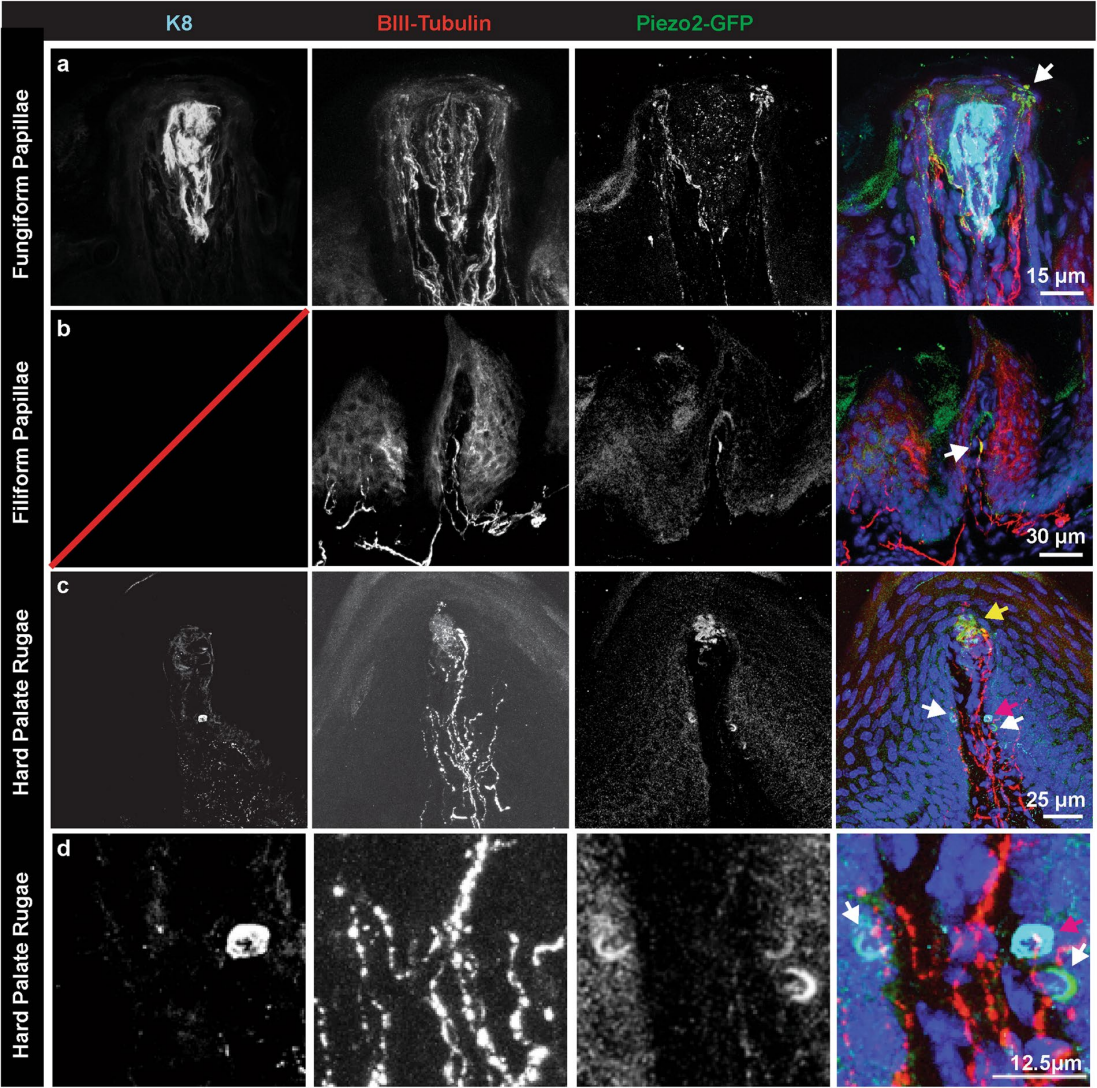
Piezo2 localizes to subsets of neuronal endings in the tongue and hard palate. (**a**) Piezo2 protein was found in sensory endings that surround taste cells in fungiform papillae (arrow). These endings form bulbous end feet that innervate the epidermis. (**b**) A subset of neuronal endings in filiform papillae were also Piezo2+. These likely represent end bulbs of Krause (arrow). (**c**) In the hard palate rugae, Piezo2 was localized to glomerular endings (yellow arrow), as well as in K8+ Merkel cells (red arrow). Piezo2+ crescent-shaped structures in the same region could represent K8- Merkel cells or adjacent sensory neuronal terminals (white arrow). (**d**) Expanded image of a Piezo2+, K8+ Merkel cell and Piezo2+, K8-.

To identify neurons in the oral cavity mediating texture detection, we analyzed protein localization of Piezo2, a mechanosensory ion channel that is required for peripheral mechanosensation^22^, using tissue from *Piezo2*^*tm1*.*1*(*Cre*)*Apat*^ (*Piezo2*-*EGFP*-*IRES*-*Cre*) mice^43^. This transgenic mouse line expresses a fusion protein of Piezo2 with EGFP, thereby expressing GFP at the precise locations where Piezo2 protein is expressed. In the fungiform papillae, Piezo2 protein is selectively localized to bulbous neuronal endings that surround K8+ taste buds (Fig. 3a), comprising a unique starburst-shaped tactile ending that innervates the epidermis of the tongue^44,45^. In filiform papillae (Fig. 3b), Piezo2 protein was located in a subset of neuronal endings in the same area where end bulbs of Krause were located (Fig. 1g), suggesting that some afferents in end bulbs of Krause are mechanosensory. In hard palate rugae, we found that Piezo2 protein localizes to both glomerular endings (Fig. 3c) as well as in crescents surrounding Merkel cells (Fig. 3c). These structures overlapped with the Merkel cell marker K8 in some cases but not others (Fig. 3c,d white and red arrows). Cells expressing Piezo2 but not K8 could be K8- Merkel cells or Merkel cell afferents^46^. These studies are the first to show a high abundance of the mechanically activated ion channel Piezo2 in the oral cavity.

### Changes in sensory architecture with aging

**Figure 4:**
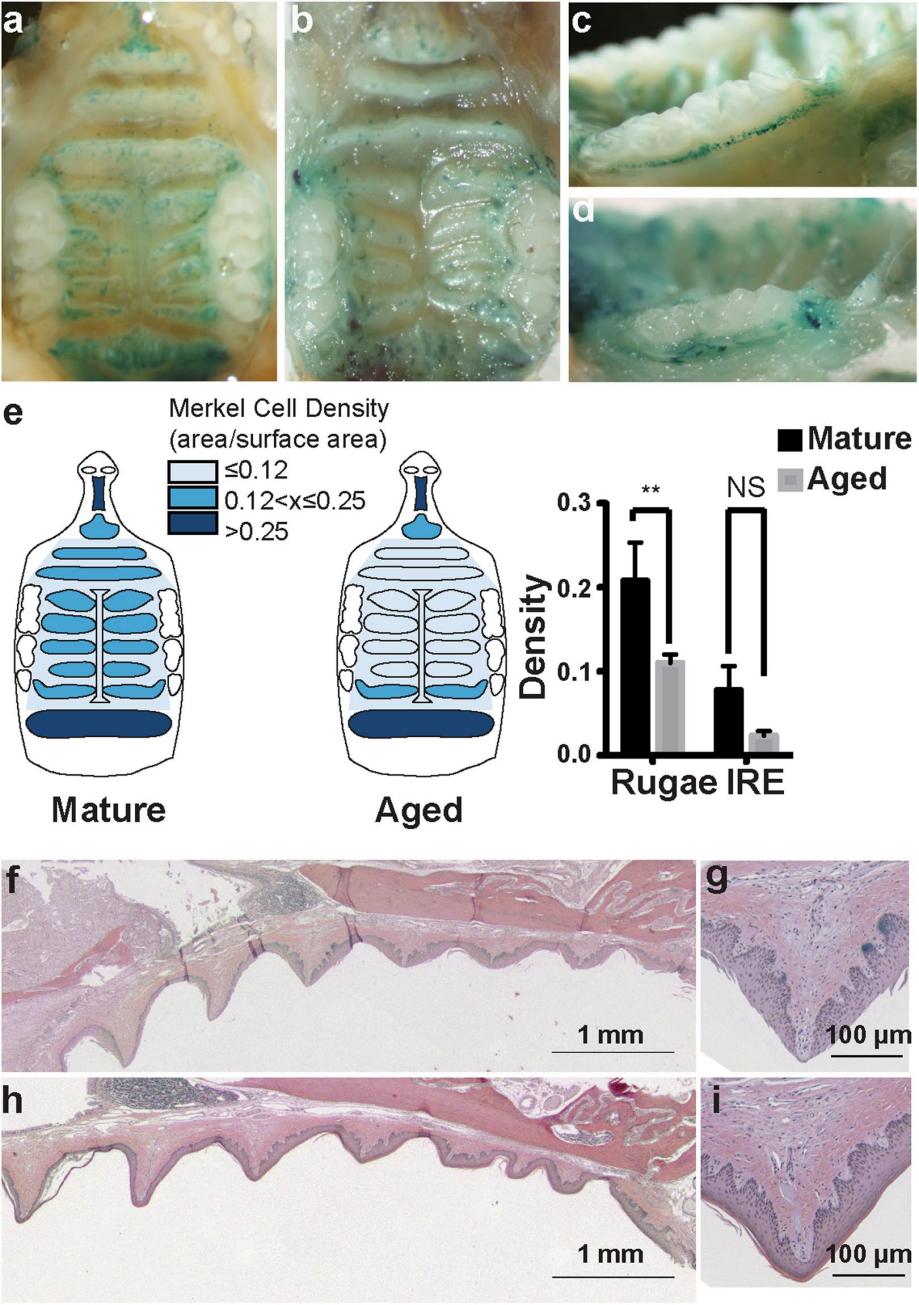
Merkel cell density reduces with age in maxillary epithelia. (**a**) *Atoh1*^*LacZ*/+^ hard palate reveals a high density of Merkel cells lining rugae. Merkel cells were particularly dense in the incisive papilla, lining the teeth, and in the postrugal field. (**b**) Aged hard palates reveal a drastic reduction in the density of Merkel cells. Note that distinct pinprick punctae are replaced with blobs of LacZ staining in some regions. (**c**) Merkel cells were found to line the maxillary gums in a discrete stripe of cells in mature mice. (**d**) In aged mice, the distinct line of Merkel cells is abolished. (**e**) Quantification of LacZ density in the hard palate reveals reductions in Merkel cells with aging (*N* = 3–4 mice). Schematics reveal decreases in the density of LacZ+ staining in antemolar and intermolar rugae. Two-way ANOVA reveals significant differences between mature (7–15 weeks) and aged (12.5–20 months) palates ( *p* = 0.0009) and between rugae and interrugal epithelium (IRE) ( *p* < 0.0001). Bonferroni posthoc analysis found significant reductions in the density of LacZ staining with age in rugal epithelium but not between IRE. ** *p*< 0.01 H&E staining of mature palate reveals structure of rugae. Blue regions are areas of LacZ+ staining. Note, stratum corneum separated during mounting process, a routine occurance in sectioning cornified tissue. (**f**) High magnification of a mature rugae shows epithelial pegs and areas of LacZ-stained epithelia. (**g**) Aged palate structure remains intact, with prominent rugae. Note reduction in blue LacZ+ regions compared with f. (**h**) High magnification of aged rugae reveals intact epithelium.

**Figure 5:**
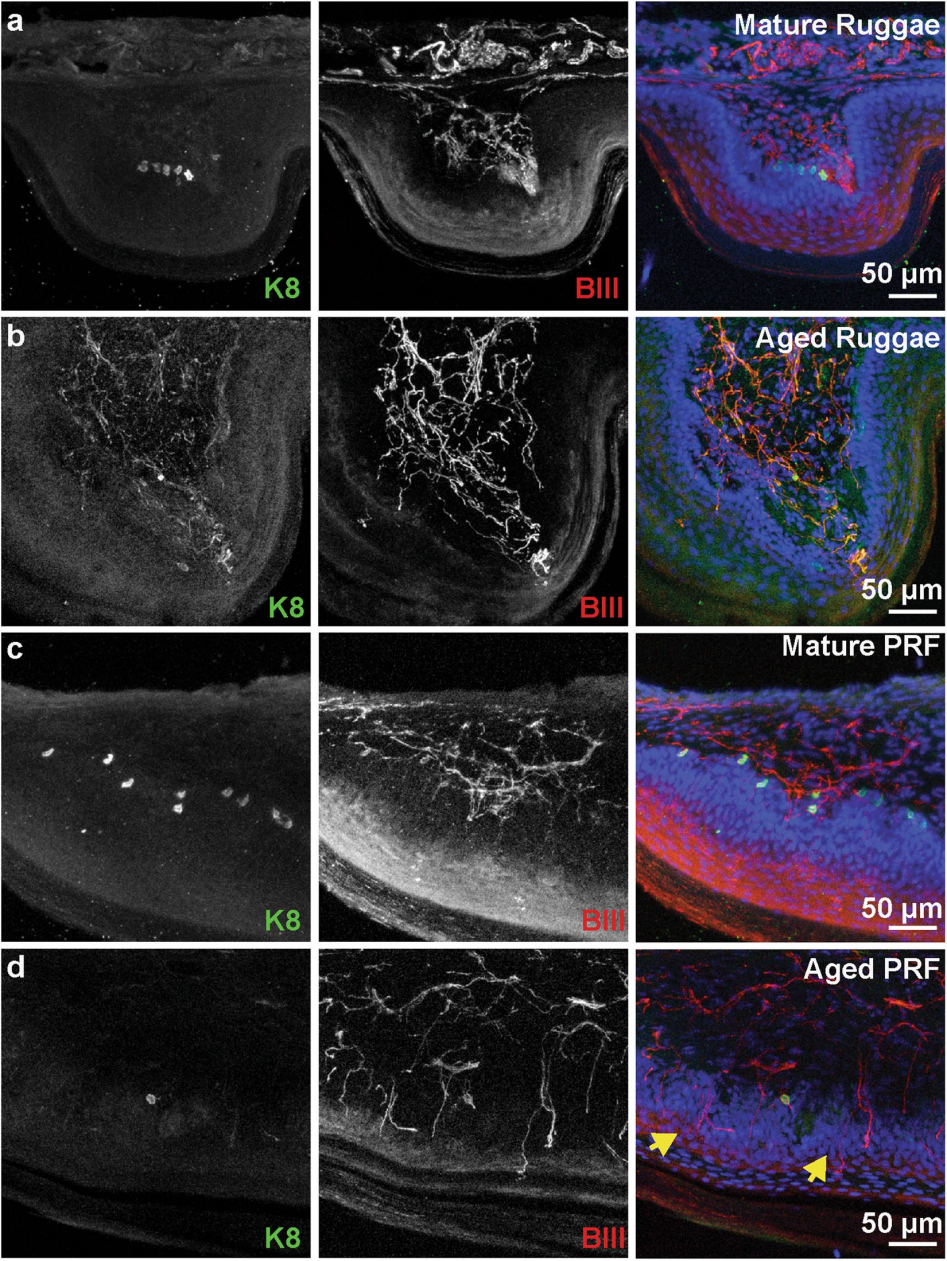
Innervation of the tongue and hard palate with age. (**a**) The posterior rugae from a mature mouse displays dense Merkel cells. (**b**) Aged posterior rugae displays no Merkel cells. (**c**) The postrugal field from a mature mouse has abundant Merkel cells, frequent neuronal endings in the lamina propria and few epidermal fibers. (**d**) The postrugal field from an aged mouse has infrequent Merkel cells, less dense neuronal innervation in the lamina propria and frequent epidermal fibers (yellow arrows).

To explore changes in sensory architecture during aging, we first analyzed the localization of Merkel cells in the hard palate and gingival mucosa. In skin, decreases in tactile sensitivity and reduced two-point discrimination are associated with aging and could be due to loss of peripheral sensory endings^47,48^. Merkel cell density declines with aging in skin^49^; however, to our knowledge no studies have shown Merkel cell loss with advanced aging in the oral cavity. Thus, we analyzed Merkel cell density in adult and aged mice. X-gal staining was performed on adult *Atoh1*^*LacZ*/+^ whole mount palates and gingiva. This method provides a comprehensive map of Merkel cell distribution and allows density calculations in whole palate and gingiva tissue. LacZ+ cells were found throughout the palatine mucosa of *Atoh1*^*LacZ*/+^ mice (Fig. 4a). In particular, LacZ+ Merkel cells were concentrated on palatine rugae, with highest densities on the incisive papilla and the postrugal field. Merkel cells were also observed at lower densities in the inter-rugal epithelium. We found a qualitative decline in the number of *Atoh1*^*LacZ*^ clusters in palates of aged mice (12–20 months, Fig. 4b). In the gingiva of mature mice, a stripe of *Atoh1*^*LacZ*^ cells lined the lateral edge of the epithelium (Fig. 4c). This stripe of Merkel cells was ablated in aged mouse gingiva (Fig. 4d). The density of X-gal staining in the palatine mucosa was quantified and revealed a significant reduction in Merkel cell density in aged compared to mature hard palates (*p* = 0.0009, Fig. 4e). Cross sections of X-gal stained hard palates revealed regions of LacZ expressing pegs in mature mice that are consistent with areas of high Merkel cell density (Fig. 4f,g). In aged mice, the overall structure of rugae remained intact (Fig. 4h,i) with fewer LacZ+ regions. To confirm the reduction in Merkel cell density using an additional histological marker, we analyzed sectioned tissue. A notable decrease in K8+ Merkel cells was identified throughout the palate (Fig. 5a–d); in agreement with global expression data from *Atoh1*^*LacZ*/+^ mice (Fig. 4). The postrugal field exhibits reorganization of neuronal fiber networks including a repartition between strata and lamina epithelial layers (Fig. 5c,d, yellow arrows). Collectively, these data suggest that mouse oral epithelia undergo modification of the neurosensory architecture with aging.

## Discussion

**Figure 6:**
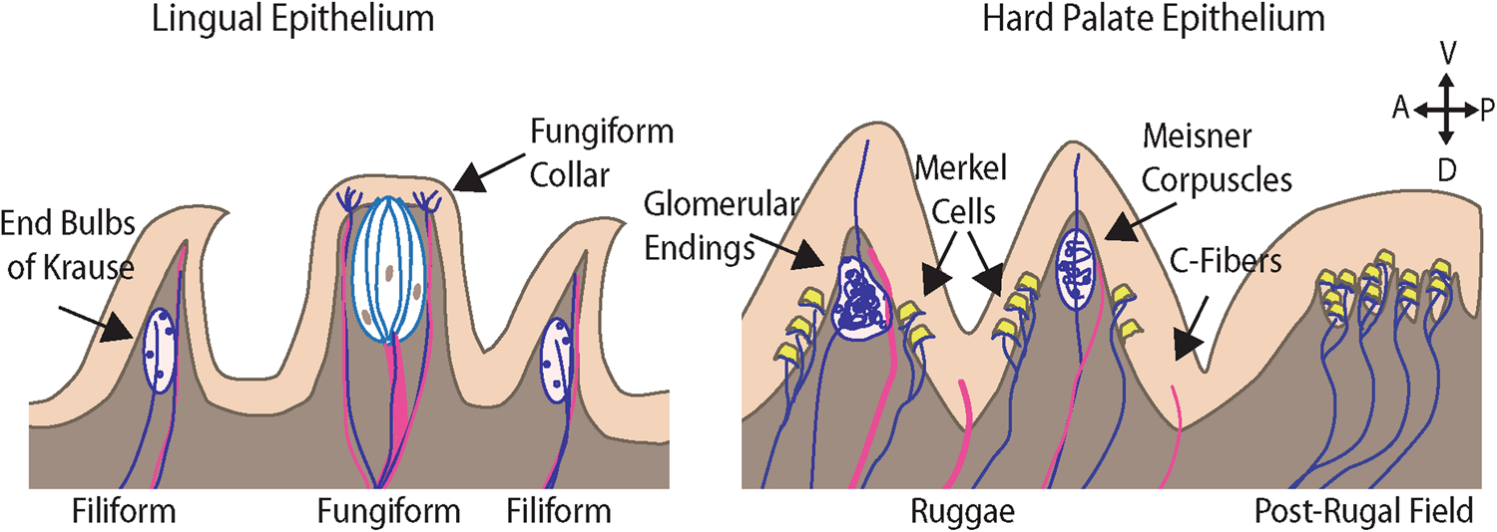
Summary of oral mechanoreceptor anatomy. Schematic of presumptive somatosensory innervation of the lingual and palatal mucosa. Myelinated afferents are shown in blue and marked by βIII tubulin and NFH; these endings are generally FM1-43+ and expected to be Piezo2+. Unmyelinated afferents are shown in pink and marked by expression of βIII tubulin and Peripherin, these are largely FM1-43- and Piezo2-. The lingual mucosa is innervated by two presumptive mechanosensory end organs. End bulbs of Krause are found in filiform papillae, while a neuronal collar surrounds taste buds in filiform papillae. In addition to these, unmyelinated afferents extend into the filiform papillae and surround taste buds. The hard palate is innervated by glomerular endings and corpuscular endings. These end organs are morphologically distinguished by the relatively disorganized appearance of glomerular endings. The hard palate rugae and post-rugal field are also densely lined with Merkel cell-neurite complexes. Finally, unmyelinated afferents are interspersed throughout the palatal epithelium. Cartoon was drawn with color schemes and symbols modeled after Fig. 1 of Bautista & Lumpkin^54^.

The oral mucosa is richly innervated with sensory afferents that play important roles in mediating feeding (Fig. 6). In this study, we investigated the diversity and distribution of sensory innervation in the oral cavity in adult and aged mice. We found that oral cavity mucosae have abundant sensory innervation. We localized expression of Piezo2, the mechanosensitive ion channel required for discriminative touch, in neuronal subtypes previously hypothesized to function in tactile sensations^22,43–45^. Furthermore, we found that Merkel cell density declines with aging, accompanied by reorganization of neuronal architecture. These results could account for alterations in a variety of oral abilities with age, including alterations in discrimination, chewing, swallowing, flavor recognition and speech production^13,50,51^.

### Neuronal distribution in lingual mucosa

Mechanoreceptors in the tongue have been hypothesized to be located in fungiform papillae, filiform papillae, or deep in muscle tissue^29,44,45^. Physiological studies on cat and human tongues have identified several classes of endings with distinct firing properties. Putative proprioceptive touch-responsive neurons have been identified that respond to high-threshold pressure, have large receptive fields and are responsive to tongue movement^52^. Neuronal moieties that are hypothesized to have superficial end-organs and roles in tactile perception include slowly adapting (SA) type I and II responses and rapidly adapting (RA) responses that either respond to pressure and stroking or stroking alone^45,52,53^. The end-organ structures that these neuronal responses correlate with have not been identified. In skin, SAI responses originate from the Merkel cell-neurite complex^54^. This is notable, considering that bona fide Merkel cells are rarely found in mammalian lingual tissue^55^. The end-organ for SAII responses is hypothesized to be Ruffini-endings, although definitive studies are still lacking^54^. Finally, RA responses in the glabrous skin are generated by Meissner’s and Pacinian corpuscles^54^.

In this study, we sought to identify somatosensory neuronal architecture in the tongue. By FM1-43 and immunohistochemistry, we found neuronal endings surrounding fungiform papillae and associated with filiform papillae. A subset of these endings express a hallmark of myelinated low-threshold mechanosensory neurons, NFH. These endings include a subset of neurons in filiform papillae and surrounding fungiform taste cells (Fig. 6). In addition to these, we also found Peripherin-expressing neurons in both of these structures that are likely nociceptors and thermoreceptors. Within filiform papillae, we identified encapsulated endings similar to end bulbs of Krause described in the cat tongue^29^. These endings were wrapped by Schwann cells, extended into connective tissue pockets embedded within individual papillae and express Piezo2 protein. Based on the morphology of these endings and their homology to Meissner’s corpuscles, we hypothesize that they are responsible for the RA currents found in physiological studies. This innervation pattern is similar to that of rats and distinct from bovine filiform papillae, where neuronal innervation is packed in connective tissue cores adjacent to keratinized papillary cones^56^. In fungiform papillae, we found a collar of neurons that extended into the epidermis surrounding taste cells. These endings have been previously shown to include both myelinated and unmyelinated subsets, to have cell bodies in the trigeminal ganglion and are hypothesized to be touch responsive^44,57^. Recent studies have also identified putative sympathetic and mechanosensory populations of neurons innervating this region from the *Phox2b* lineage^58,59^. We find that these endings express both NFH and Piezo2 and label with FM1-43, lending strong weight to a role in mechanosensitivity for a subset of these neurons. This finding is surprising, as neuronal endings that innervate the epidermis are not usually low-threshold mechanoreceptors, but rather unmyelinated C-fibers. Thus, the response properties of these neurons and whether they contribute to the SA responses remains an open question.

### Innervation of the maxillary mucosa

Investigations into the innervation of hard palate and gingiva have identified several distinct end-organ structures. In the hard palate, high densities of Merkel cells have been reported in rugal epithelium^27,30^. Corpuscular endings have been described in detail in the mammalian hard palate including Meissner’s, Pacinian and Ruffini endings^25,27^. The hard palate is unique compared to other epithelial tissues in that corpuscular endings have been identified with apical neuronal processes that extend into the superficial layers of the epithelium, deemed ultra-terminals^41^. Free nerve endings have been identified in both basal and superficial layers of the epithelium^60^. In the gingiva, previous studies have found Merkel cells and Meissner’s corpuscles throughout the epithelium^26,61–63^.

We have confirmed and extended these previous findings in the hard palate epithelium. We observed endings that resemble Meissner’s and glomerular corpuscles^30^ (Fig. 6). In contrast to primates, we did not find definitive Pacinian or Ruffini corpuscles in this study^25^. Remarkably, we found frequent ultra-terminals in both whole mount FM1-43 studies and in section immunohistochemistry. These structures are believed to be extensions of corpuscular neuronal afferents rather than C-fibers, as low-density staining reveals these are extensions of corpuscular end organs rather than free endings^41^. In addition to this, ultra-terminals were identified that are FM1-43+ and NFH+, features that are not typical of c-fibers. It is also possible, that these endings could act as rapidly adapting touch receptors, as they share anatomical homology to the epidermal free nerve endings found in the Eimer’s organ of the star-nosed mole^64,65^. Currently, the function of these endings is completely unknown and a matter of speculation as to whether these modulate touch sensitivity or perhaps integrate temperature or chemical sensitivity to these afferents. As in previous reports, we find that Merkel cells are most dense on the palatine rugae with lower densities in the inter-rugal epithelium^27^. We extend these findings by providing a quantitative map of Merkel cell density in the hard palate. The highest densities of Merkel cells were found in the incisive papilla and the post-rugal field. Of these hard palate end organs, corpuscular endings and Merkel cells express Piezo2 protein. In addition to these, K8- cells in the epithelium also express Piezo2. Based on the location of these cells, they are likely Merkel cells that are not fully mature or SAI afferent endings. Additional analyses are needed to disentangle the molecular heterogeneity and linage of oral cavity Merkel cells.

Reports on the distribution, density and innervation of Merkel cells in the gingival mucosa are conflicting. Aimetti and colleagues found few Merkel cells in human gums with little innervation^61^. Others have found high densities of Merkel cells in human and primate gums^28,66,67^, but with sparse innervation. A possible explanation for these discrepancies could be sampling errors due to the tendency of Merkel cells to be localized in clusters^26^, or due to variations in the population due to age and injuries to the gums. In this work, we find a high density of Merkel cells in the adult mouse gingival mucosa that are contacted by βIII+ neuronal afferents. Interestingly, we could not identify encapsulated corpuscles in the maxillary gingiva, as previously found in human and rat tissues^62,63^. These results could reflect inter-species differences, or differences in techniques applied (e.g. differences in antigen specificity or immunohistochemistry vs. electron microscopy).

Collectively, the organization of the palatine mucosa resembles that of the high-acuity sensing rete ridges of the fingertip with encapsulated corpuscles jutting up in epithelial pegs of ridges and Merkel cells positioned at the base (Fig. 6). The mouse maxillary gingival mucosa is primarily lined with Merkel cell-neurite complexes. These are both in contrast to the lingual mucosa, that is innervated by encapsulated endings embedded in conical shaped filiform papillae and a novel NFH+ neuronal ending that extends into epithelial layers. How these variety of neuronal endings contribute to somatosensation necessary for tissue-specific functions has yet to be determined.

### Changes in sensory architecture with aging

Aged populations are at higher risk for a multitude of adverse oral health issues with potential somatosensory abnormalities. These include decreased chewing, swallowing, speech, tactile sensitivity as well as burning mouth syndrome^13,51,68^. Multiple parameters of sensory function have been found to decline with age that may be related to symptomology; however, changes in sensorineural architecture that mediate these effects remain elusive. In this work, we have identified profound alterations in mechanosensory architecture in the oral cavity in aged mice. We found that Merkel cells, a class of pressure sensitive sensory cells, undergo a drastic decline with age. Age related alterations in Meissner’s corpuscle architecture are well documented in cutaneous and oral tissues^20,69^; yet this study is the first to report coincident reductions in Merkel cell density. Notably, these changes are associated with decreased feeding abilities in mice^70^. Whether reduced tactile abilities could influence feeding behaviors is open to further investigation. Furthermore, how different mechanosensory submodalities influence alterations in specific oral functions are yet to be determined.

In summary, this work has provided an immunohistochemical map of somatosensory receptors in the oral cavity including the tongue, hard palate and gingiva. We found that a subset of these neurons express Piezo2, a principle mediator of mechanosensitivity, and that these neurons label with FM1-43, providing strong evidence that they are tactile receptors. We then found that Merkel cells undergo a dramatic reduction in density with aging coincident with reduction in encapsulated endings and feeding abilities in rodents.

## Materials and Methods

### Animal Use

All animal experiments were approved by Columbia University’s Institutional Animal Care and Use Committee (IACUC) and conducted in accordance with Columbia University’s IACUC Policies.

Experiments were conducted using either C57BL/6J (Jackson Labs) or transgenic mouse lines. Transgenic mouse lines were kept on mixed genetic backgrounds. *Atoh1*^*LacZ*^ mice (MGI: *Atoh1*^*tm2Hzo*^)^38^ were used for comprehensively mapping Merkel cells at low-resolution. In the *Atoh1*^*LacZ*^ line, the *Atoh1* coding region is replaced with *LacZ*, resulting in β-galactosidase expression in cells where *Atoh1* is normally expressed. The *Atoh1*^*tm4*.*1Hzo*^ mouse line^39^, which expresses an Atoh1-enhanced green fluorescent protein (EGFP) fusion protein, were used for detailed histology of Merkel cell distributions in sections. In addition, wild-type littermates were used to verify specificity of marker expression. To localize Piezo2 protein in tissues we employed mice that harbor a targeted allele with EGFP fused to the Piezo2 protein (*Piezo2*^*EGFP*-*IRES*-*Cre*^; MGI: *Piezo2*^*tm1*.*1*(*cre*)*Apat*^)^43^.

Mature mice used in this study were 7–15 weeks old. Aged mice were 12.5–20 months old. Both male and female mice were used in all experiments. For all histology experiments N ≥ 2 independent mice.

### Tissue Processing and Histology

All experiments were performed at room temperature unless otherwise noted.

X-gal staining was used for β-galactosidase detection. Tongue, palate and gum specimens were dissected from *Atoh1*^*LacZ*/+^ and WT control animals. Tissue was fixed for 20 min in 4% paraformaldehyde (PFA) and then washed with phosphate buffered saline (PBS). Tissue was then rinsed three times in PBS containing 0.02% NP40 and 0.01% deoxycholate. Staining was performed overnight at 37 °C on a rotary shaker in PBS containing 5 mM K_3_Fe(CN)_6_, 5 mM K_4_Fe(CN)_6_, 0.02% NP40, 0.01% deoxycholate, 2 mM MgCl_2_, 5 mM EGTA, and 1 mg/ml X-gal. After staining, tissue was post-fixed in 4% PFA for 10 min.

For immunohistochemistry in tissue cryosections, tongue specimens were flash frozen in Tissue-Tek OCT over liquid nitrogen. Palate, gum, and teeth tissue was fixed for 2 h in 4% PFA at room temperature, washed in PBS and then decalcified for 1–2 weeks in 10% EDTA pH 7.4 at 4 °C on a rotary mixer. When soft, tissue was cryoprotected in 30% sucrose overnight at 4 °C and then embedded in Tissue-Tek OCT over liquid nitrogen. Sections (25 µm) were taken using a cryostat onto Superfrost slides (Fisherbrand).

For hematoxylin and eosin (H&E) staining, specimens were fixed overnight in Formalde-Fresh (10% formalin). Bony tissue was decalcified in 10% EDTA as described above. Tissues were then dehydrated in an ethanol series, cleared in xylene and embedded in paraffin blocks. Sections (5 µm) were cut and stained as previously described^71^.

For immunohistochemistry, slides were baked 45 min to 3 h at 37 °C to ensure adhesion and prehybridized in PBS containing 5% Normal goat serum and 0.3% Triton-X 100. Slides were then hybridized overnight at 4 °C with primary antibodies (Table 1) diluted in hybridization solution. Slides were then washed three times in PBS with 0.3% Triton-X 100, incubated with secondary antibodies diluted in hybridization buffer for 45 min to 2 h, washed three times in PBS and embedded in Fluoromount-G with DAPI (Southern Biotech).

**Table 1:** Antibodies used in this study.

Antibody	Cells marked	Source	Dilution	Catalog #	Lot #
Chicken anti-GFP	GFP expressing Merkel cells	Abcam	1:1000	ab13970	GR89472-25 GR53074-1
Rat anti-Keratin 8	Merkel cells & taste cells	DSHB	1:100	TROMA1-s	12/31/14–22 µg/ml 7/7/16–30 µg/ml
Chicken anti-Neurofilament H	Myelinated neurons	Abcam	1:2000	ab4680	GR174538-12 GR260044-11
Rabbit anti-Neurofilament H	Myelinated neurons	Abcam	1:1000	Ab8135	GR261841-4
Chicken anti-βIII tubulin	All neurons	Abcam	1:3000	ab107216	GR250436-1
Rabbit anti-βIII tubulin	All neurons	Abcam	1:2000	ab18207	GR204562-1 GR220660-1
Chicken anti-Nestin	Schwann cells	Novus	1:200	NB100-1604	NES-89707987 0407
Rabbit anti S100	Terminal Schwann cells	Dako	1:100	20311	00084964
Rabbit anti-Peripherin	Small diameter neurons	Thermo Scientific	1:500	PA3-16723	PG1878101E RB2152015

### FM1-43 injections

Mice were injected with 2–3 mg/kg FM1-43 (Synaptogreen C4, Biotium Catalog #70022). After 12–16 hours, tongues were removed, and the epithelium was dissected and laid flat between coverslips with Fluoromount-G mounting media.

### Imaging, Equipment and Settings

Immunohistochemistry images were acquired on laser scanning confocal microscope (Carl Zeiss LSR Exciter) equipped with a 20X 0.8 NA and 40X 1.3 NA objective lenses. Images were taken at 1024 × 1024 pixels with two line averages. For 20X images, pixel sizes were 0.31 µm with a pixel dwell of 1.28 µs. For 40X images, pixel sizes were 0.16 µm with a pixel dwell time of 1.6 µs. Merkel cell density and depth mapping images were acquired on a Zeiss Axioplan2 microscope using a 10X 0.45 NA objective lens. Exemplary LacZ images were acquired on an Olympus SZX16 microscope. Brightfield imaging was performed on an AxioObserver Z.1 microscope (Zeiss, Thornwood, NY) using a 10x/0.5 or 2.5x/0.075 objective lens and an Axiocam ICc camera (Zeiss). Images were collected and stitched, where applicable, using ZEN software (Zeiss). Images were processed using ImageJ, Adobe Photoshop and Adobe Illustrator. Minor adjustments for brightness, contrast and threshold were made in Adobe Photoshop and applied to the entire image. Some images were smoothed with a Gaussian blur of 0.5–1.0 pixel. Densitometry was conducted using ImageJ software. LacZ positive areas were compared with total areas using the ROI function. Data were analyzed using Graphpad Prism.

### Data availability

The datasets generated during and/or analyzed during the current study are available from the corresponding author on reasonable request.

## Electronic supplementary material

Supplemental Information

Supplemental movie 1

## References

[1] Mouritsen OG (2016). Gastrophysics of the Oral Cavity. Curr Pharm Des.

[2] Mars M, Hogenkamp PS, Gosses AM, Stafleu A, De Graaf C (2009). Effect of viscosity on learned satiation. Physiol Behav.

[3] Dominy NJ (2016). How chimpanzees integrate sensory information to select figs. Interface Focus.

[4] Zijlstra N, Mars M, de Wijk RA, Westerterp-Plantenga MS, de Graaf C (2008). The effect of viscosity on ad libitum food intake. Int J Obes (Lond).

[5] Zijlstra N (2009). Effect of viscosity on appetite and gastro-intestinal hormones. Physiol Behav.

[6] Peyron MA, Mishellany A, Woda A (2004). Particle size distribution of food boluses after mastication of six natural foods. J Dent Res.

[7] Hutchings JB, Lillford PJ (1988). The Perception of Food Texture - the Philosophy of the Breakdown Path. J Texture Stud.

[8] Le Reverend, B. & Hartmann, C. Numerical modeling of human mastication, a simplistic view to design foods adapted to mastication abilities. *Physiol Behav*, 10.1016/j.physbeh.2013.10.012 (2013). 24471180

[9] Le Reverend B, Saucy F, Moser M, Loret C (2016). Adaptation of mastication mechanics and eating behaviour to small differences in food texture. Physiol Behav.

[10] Steele CM, Miller AJ (2010). Sensory input pathways and mechanisms in swallowing: a review. Dysphagia.

[11] Boffano P, Roccia F, Gallesio C (2012). Lingual nerve deficit following mandibular third molar removal: review of the literature and medicolegal considerations. Oral Surg Oral Med Oral Pathol Oral Radiol.

[12] Brill N, Tryde G, Edwards C, Thomas H (1974). Age changes in the two-point discrimination threshold in human oral mucosa. J Oral Rehabil.

[13] Teranaka S, Shibaji T, Minakuchi S, Uematsu H (2008). Age-related changes in oral mechanosensitivity of symptom-free subjects. J Med Dent Sci.

[14] Fucci D, Petrosino L (1983). Lingual vibrotactile sensation magnitudes: comparison of suprathreshold responses for three different age ranges. Percept Mot Skills.

[15] Ikebe K (2007). Association between oral stereognostic ability and masticatory performance in aged complete denture wearers. Int J Prosthodont.

[16] Kawagishi S, Kou F, Yoshino K, Tanaka T, Masumi S (2009). Decrease in stereognostic ability of the tongue with age. J Oral Rehabil.

[17] Grasso JE, Catalanatto FA (1979). The effects of age and full palatal coverage on oral stereognostic ability. J Prosthet Dent.

[18] Landt H, Fransson B (1975). Oral ability to recognize forms and oral muscular coordination ability in dentulous young and elderly adults. J Oral Rehabil.

[19] Nedelman C, Bernick S (1981). Changes in nerve supply to aging human gingiva. J Prosthet Dent.

[20] Iida S, Tachibana T (1996). Age-related changes in Meissner corpuscles in the mouse palate: a histochemical and ultrastructural study. Archives of histology and cytology.

[21] Johnson KO, Yoshioka T, Vega-Bermudez F (2000). Tactile functions of mechanoreceptive afferents innervating the hand. J Clin Neurophysiol.

[22] Ranade SS (2014). Piezo2 is the major transducer of mechanical forces for touch sensation in mice. Nature.

[23] Meyers JR (2003). Lighting up the senses: FM1-43 loading of sensory cells through nonselective ion channels. The Journal of neuroscience: the official journal of the Society for Neuroscience.

[24] Goldstein ME, House SB, Gainer H (1991). NF-L and peripherin immunoreactivities define distinct classes of rat sensory ganglion cells. J Neurosci Res.

[25] Halata Z, Baumann KI (1999). Sensory nerve endings in the hard palate and papilla incisiva of the rhesus monkey. Anatomy and embryology.

[26] Kingsmill VJ, Berkovitz BK, Barrett AW (2005). An immunohistochemical analysis of human Merkel cell density in gingival epithelium from dentate and edentulous subjects. Archives of oral biology.

[27] Nunzi MG, Pisarek A, Mugnaini E (2004). Merkel cells, corpuscular nerve endings and free nerve endings in the mouse palatine mucosa express three subtypes of vesicular glutamate transporters. Journal of neurocytology.

[28] Righi A (2006). Merkel cells in the oral mucosa. International journal of surgical pathology.

[29] Spassova I (1974). Ultrastructure of the simple encapsulated nerve endings (simple end-bulbs of Krause) in the tongue of the cat. Journal of anatomy.

[30] Tachibana T, Fujiwara N, Sato H, Nawa T (1990). A comparative electron microscopic analysis of mechanoreceptors in the hard palate of the mouse (Mus musculus; Rodentia) and the musk shrew (Suncus murinus; Insectivora). Archives of oral biology.

[31] Tachibana T (1997). Polymorphism of Merkel cells in the rodent palatine mucosa: immunohistochemical and ultrastructural studies. Archives of histology and cytology.

[32] Yoshie S, Yokosuka H, Kanazawa H, Fujita T (1999). The existence of Merkel cells in the lingual connective tissue of the Surinam caiman, Caiman crocodilus crocodilus (order Crocodilia). Archives of histology and cytology.

[33] Toyoshima K, Shimamura A (1991). Uranaffin reaction of Merkel corpuscles in the lingual mucosa of the finch, Lonchula striata var. domestica. Journal of anatomy.

[34] Toyoshima K, Seta Y, Toyono T, Takeda S (1999). Merkel cells are responsible for the initiation of taste organ morphogenesis in the frog. The Journal of comparative neurology.

[35] Toyoshima K, Miyamoto K, Itoh A, Shimamura A (1987). Merkel-neurite complexes in the fungiform papillae of two species of monkeys. Cell Tissue Res.

[36] Maricich SM (2009). Merkel cells are essential for light-touch responses. Science.

[37] Ben-Arie N (2000). Functional conservation of atonal and Math1 in the CNS and PNS. Development.

[38] Bermingham NA (1999). Math1: an essential gene for the generation of inner ear hair cells. Science.

[39] Rose MF (2009). Math1 is essential for the development of hindbrain neurons critical for perinatal breathing. Neuron.

[40] Asano-Miyoshi M, Hamamichi R, Emori Y (2008). Cytokeratin 14 is expressed in immature cells in rat taste buds. J Mol Histol.

[41] Gairns FW (1954). Sensory nerve endings in the human palate. J Physiol.

[42] Ichikawa H, Matsuo S, Silos-Santiago I, Sugimoto T (2000). Developmental dependency of Meissner corpuscles on trkB but not trkA or trkC. Neuroreport.

[43] Woo SH (2014). Piezo2 is required for Merkel-cell mechanotransduction. Nature.

[44] Whitehead MC, Beeman CS, Kinsella BA (1985). Distribution of taste and general sensory nerve endings in fungiform papillae of the hamster. Am J Anat.

[45] Robinson PP (1992). The effect of injury on the properties of afferent fibres in the lingual nerve. The British journal of oral & maxillofacial surgery.

[46] Marshall KL (2016). Touch Receptors Undergo Rapid Remodeling in Healthy Skin. Cell Rep.

[47] Wickremaratchi MM, Llewelyn JG (2006). Effects of ageing on touch. Postgrad Med J.

[48] Stevens JC, Choo KK (1996). Spatial acuity of the body surface over the life span. Somatosens Mot Res.

[49] Lumpkin EA (2003). Math1-driven GFP expression in the developing nervous system of transgenic mice. Gene expression patterns: GEP.

[50] Brownie S (2006). Why are elderly individuals at risk of nutritional deficiency?. Int J Nurs Pract.

[51] Mioche L, Bourdiol P, Peyron MA (2004). Influence of age on mastication: effects on eating behaviour. Nutr Res Rev.

[52] Trulsson M, Essick GK (1997). Low-threshold mechanoreceptive afferents in the human lingual nerve. J Neurophysiol.

[53] Biedenbach MA, Chan KY (1971). Tongue mechanoreceptors: comparison of afferent fibers in the lingual nerve and chorda tympani. Brain research.

[54] Bautista DM, Lumpkin EA (2011). Perspectives on: information and coding in mammalian sensory physiology: probing mammalian touch transduction. J Gen Physiol.

[55] Lacour JP, Dubois D, Pisani A, Ortonne JP (1991). Anatomical mapping of Merkel cells in normal human adult epidermis. The British journal of dermatology.

[56] Sato O, Maeda T, Kobayashi S, Iwanaga T, Fujita T (1988). Filiform papillae as a sensory apparatus in the tongue: an immunohistochemical study of nervous elements by use of neurofilament protein (NFP) and S-100 protein antibodies. Cell Tissue Res.

[57] Beidler, L. M. Innervation of Rat Fungiform Papilla. *Olfaction and Taste* (eds Carl Pfaffman, *International Congress of Physiological Sciences,* & *International Symposium of Olfaction and Taste*) 352–369 (Rockefeller University Press, 1968).

[58] Ohman-Gault L, Huang T, Krimm R (2017). The transcription factor Phox2b distinguishes between oral and non-oral sensory neurons in the geniculate ganglion. The Journal of comparative neurology.

[59] Donnelly CR, Shah AA, Mistretta CM, Bradley RM, Pierchala BA (2018). Biphasic functions for the GDNF-Ret signaling pathway in chemosensory neuron development and diversification. Proc Natl Acad Sci USA.

[60] Yeh Y, Byers MR (1983). Fine structure and axonal transport labeling of intraepithelial sensory nerve endings in anterior hard palate of the rat. Somatosensory research.

[61] Aimetti M (2010). Merkel cells and permanent disesthesia in the oral mucosa after soft tissue grafts. Journal of cellular physiology.

[62] Lewinsky, W. & Stewart, D. The Innervation of the Human Gum. *Journal of anatomy* **72**, 531–534 533 (1938). PMC1252360 17104724

[63] Martinez R, Pekarthy JM (1974). Ultrastructure of encapsulated nerve endings in rat gingiva. 1. Am J Anat.

[64] Marasco PD, Tsuruda PR, Bautista DM, Julius D, Catania KC (2006). Neuroanatomical evidence for segregation of nerve fibers conveying light touch and pain sensation in Eimer’s organ of the mole. Proc Natl Acad Sci USA.

[65] Marasco PD, Catania KC (2007). Response properties of primary afferents supplying Eimer’s organ. J Exp Biol.

[66] Ramieri G (1992). Non-innervated Merkel cells and Merkel-neurite complexes in human oral mucosa revealed using antiserum to protein gene product 9.5. Archives of oral biology.

[67] Turner DF (1983). The morphology and distribution of Merkel cells in primate gingival mucosa. The Anatomical record.

[68] Kohorst JJ, Bruce AJ, Torgerson RR, Schenck LA, Davis MD (2015). The prevalence of burning mouth syndrome: a population-based study. The British journal of dermatology.

[69] Bolton CF, Winkelmann RK, Dyck PJ (1966). A quantitative study of Meissner’s corpuscles in man. Neurology.

[70] Lever TE (2015). Videofluoroscopic Validation of a Translational Murine Model of Presbyphagia. Dysphagia.

[71] Fantauzzo KA, Kurban M, Levy B, Christiano AM (2012). Trps1 and its target gene Sox9 regulate epithelial proliferation in the developing hair follicle and are associated with hypertrichosis. Plos genetics.

