# Supplementary material for: Somatosensory innervation of the oral mucosa of adult and aging mice
Source: Sci Rep. 2018 Jul 2;8:9975. doi: 10.1038/s41598-018-28195-2 (PMC6028454; doi:10.1038/s41598-018-28195-2)
Supplement: Supplementary file 1 — Supplemental Information [file 41598_2018_28195_MOESM1_ESM.pdf]

Supplemental Information

Somatosensory innervation of the oral mucosa of adult and aging mice

Yalda Moayedi, Lucia Duenas-Bianchi, Ellen A. Lumpkin

Supplemental Movie 1. FM1-43 labelling of a fungiform papillae reveals net of neurons surrounding the taste bud.

Optical sections through the depth of the fungiform papillae shows neurons surround the taste bud.

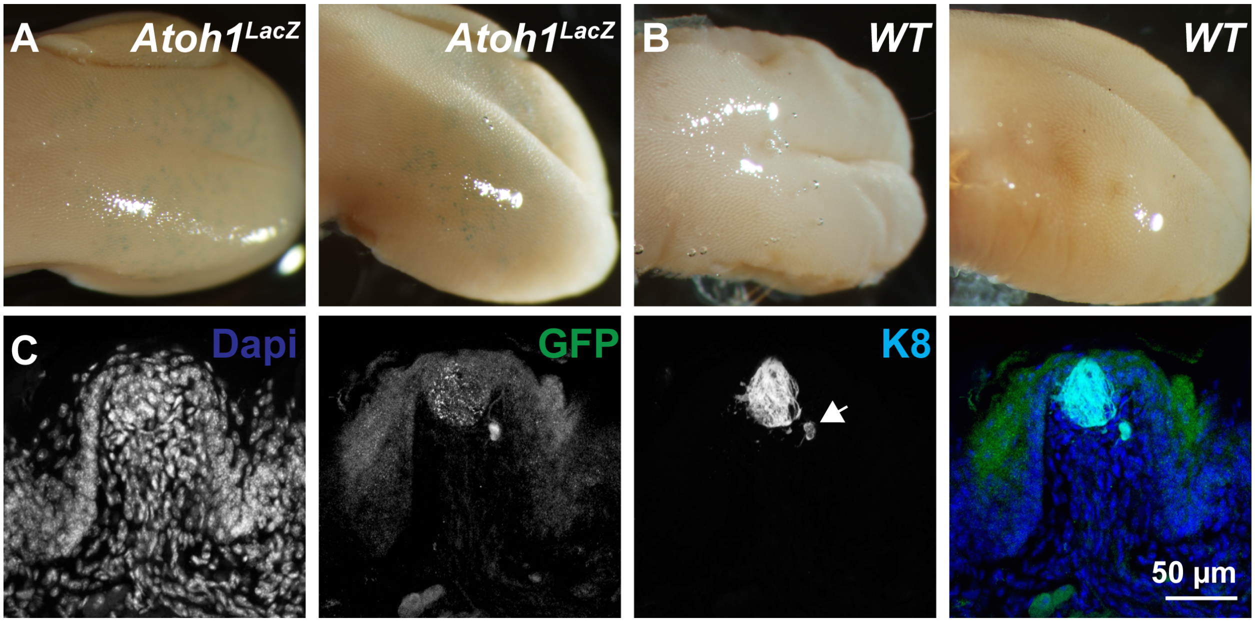

Supplemental Figure 1. Merkel cells are sparse, if not absent, in the mouse lingual epithelium.

- **A.** X-gal staining of *Atoh1*$^{LacZ/+}$ tongues reveals LacZ+ puncta
- **B.** LacZ+ puncta are absent in *WT* littermates is shown.
- **C.** Tongue sections were screened using a genetic Atoh1 reporter to identify putative Merkel cells. In three tongues, we identified a single Atoh1+ K8+ cell adjacent to taste bud, that expresses molecular markers of Merkel cells but has an atypical morphology (arrow).
